# Selfies of Imperial Cormorants (*Phalacrocorax atriceps*): What Is Happening Underwater?

**DOI:** 10.1371/journal.pone.0136980

**Published:** 2015-09-14

**Authors:** Agustina Gómez-Laich, Ken Yoda, Carlos Zavalaga, Flavio Quintana

**Affiliations:** 1 Instituto de Biología de Organismos Marinos (IBIOMAR-CENPAT), Consejo Nacional de Investigaciones Científicas y Técnicas (CONICET), Boulevard Brown 2915, Puerto Madryn (U9120ACD), Chubut, Argentina; 2 Graduate School of Environmental Studies, Nagoya University, Furo-cho, Chikusa-ku, Nagoya 464–8601, Japan; 3 Facultad de Ciencias Ambientales, Universidad Científica del Sur, Carretera Antigua, Panamericana Sur km 19, Lima 42, Perú; 4 Wildlife Conservation Society, Amenabar 1595, (C1426AKC), Ciudad de Buenos Aires, Argentina; Hawaii Pacific University, UNITED STATES

## Abstract

During the last few years, the development of animal-borne still cameras and video recorders has enabled researchers to observe what a wild animal sees in the field. In the present study, we deployed miniaturized video recorders to investigate the underwater foraging behavior of Imperial cormorants (*Phalacrocorax atriceps*). Video footage was obtained from 12 animals and 49 dives comprising a total of 8.1 h of foraging data. Video information revealed that Imperial cormorants are almost exclusively benthic feeders. While foraging along the seafloor, animals did not necessarily keep their body horizontal but inclined it downwards. The head of the instrumented animal was always visible in the videos and in the majority of the dives it was moved constantly forward and backward by extending and contracting the neck while travelling on the seafloor. Animals detected prey at very short distances, performed quick capture attempts and spent the majority of their time on the seafloor searching for prey. Cormorants foraged at three different sea bottom habitats and the way in which they searched for food differed between habitats. Dives were frequently performed under low luminosity levels suggesting that cormorants would locate prey with other sensory systems in addition to sight. Our video data support the idea that Imperial cormorants’ efficient hunting involves the use of specialized foraging techniques to compensate for their poor underwater vision.

## Introduction

The foraging behavior of seabirds has been difficult to study, principally because they spend most of their time at sea and obtain their food underwater. Fortunately, over the last 30 years, technological advances have resulted in the development of recording devices that can be attached to marine animals, providing information about their movements and behaviors at sea (e.g. [1, 2, 3]). Moreover, using devices that can record behavioral and environmental parameters simultaneously, researchers have been able to collect data on not only animal movement and behavior but also on the physical characteristics (e.g., temperature, salinity) of the marine environment [1, 4, 5, 6]. However, all these devices only provide indirect information in the form of electronic signals and do not allow direct observation of the behavior or the environment visited by the tracked animal.

During the last few years, development of animal-borne still cameras and video recorders has enabled researchers to observe what a wild animal sees in the field (e.g. [7, 8, 9]). Used in conjunction with other electronic tracking equipment, such as GPS tracking recorders and accelerometers [9, 10, 11, 12], these devices can provide unique insights into multiple aspects of animal behavior and ecology. Some examples of the numerous applications of camera technology include studies on prey capture tactics [13, 14]; intra- and inter-species interactions [12, 15, 16, 17]; characteristics of the habitats where predators forage [18]; interactions between the tracked subjects and the environment [12]; and even information about contact rates, which can be used to understand the potential for disease transmission [19]. However, the main disadvantage of current animal-borne cameras is the high power consumption and large storage space, which limit the recording period [9].

Imperial cormorants (*Phalacrocorax atriceps*) from Punta León (Patagonia, Argentina) forage benthically [20, 21] and principally consume cusk-eels (*Raneya fluminensis*), benthic fish (*Riberoclinus eigenmanni*), and toadfish (*Thiathalassothia argentina*) throughout the breeding season [22] (Harris et al. unpublished data). Previous studies using tri-axial accelerometers on Imperial cormorants have reported an accurate record of their movement patterns [23] and also enabled the determination of a precise foraging trip time budget [21]. Moreover, simultaneous use of accelerometers and beak opening angle sensors has resulted in a better understanding of how much time cormorants spend searching for and pursuing prey, as well as how buoyancy might affect prey-capture performance [20]. Despite these advances, little is known about cormorants´ hunting techniques (but see [24]), and if prey capture tactics change with the type of microhabitat visited. Another issue that remains unclear is how visual hunters like cormorants locate prey on dives performed during the night [25] or to water depths of more than 100 m [26]. The general purpose of the present study was to investigate the underwater foraging behavior of Imperial cormorants by using bird-borne video cameras. Our specific aims were: (1) to determine the underwater activities performed by cormorants and complement the information previously obtained by the use of accelerometers and time-depth recorders, (2) to give a general description of the microhabitats used by Imperial cormorants and determine if the underwater foraging strategy varies according to the microhabitat visited, and (3) to examine the role of underwater vision in prey detection. Imperial cormorants are a useful model to study underwater foraging behavior and microhabitat use for the following reasons. First, these birds are generally described as visual hunters, performing dives during the day in the euphotic zone ([24, 27] but see [25]). Second, during the breeding season, Imperial cormorants show a sex-specific timing of foraging with females departing right after dawn and males leaving by noon [28], so it is possible to instrument a bird just before it goes to sea maximizing the use of the camera battery.

## Materials and Methods

**Fig 1:**
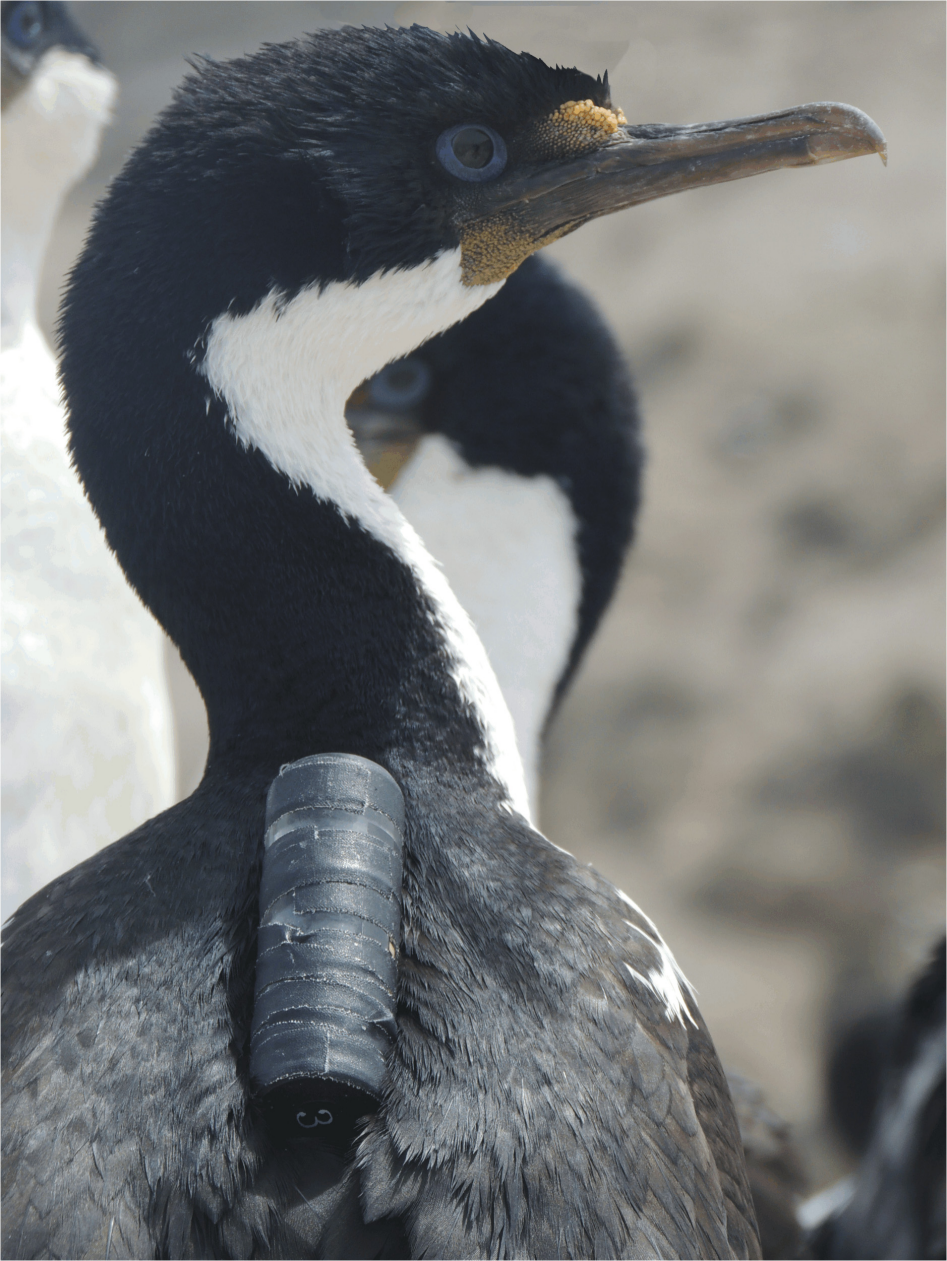
Male Imperial Cormorant (*Phalacrocorax atriceps*) instrumented with a water resistant video camera. Photograph illustrating a camera attached to the upper back feathers of a bird.

Fieldwork was conducted at Punta León provincial protected area (43°04′S; 64°29′W) in December 2011 and 2012 under the project “"Ecología pelágica de aves marinas buceadoras: determinación de movimientos y comportamiento en el mar mediante la utilización de registradores electrónicos de alta resolución" directed by Flavio Quintana and authorized by La Secretaria de Turismo y Áreas Protegidas (STyAP) and La Dirección de Fauna y Flora Silvestre (DFyFS) of the Province of Chubut. Thirty-three male breeding Imperial cormorants were instrumented with 12 POV MAC10 Mini waterproof action video cameras designed to be water resistant at depth < 10 m (6.5 cm in length, 1.5 cm in diameter, 20.5 g, 736 × 480 pixels, 25 frames per second). Because Imperial cormorants dive deeper than 10 m, the cameras were protected in underwater custom housings (8 cm in length and 2.5 cm in diameter) (Nautilus, Tail, Yokohama, Japan). The total weight of the camera plus the housing was 49.3 g, less than 2.2% of the average adult male body weight (2.3 kg) [29]. Each cormorant was slowly removed from its nest using a specially designed hook. The hook consisted of a crook at the end of a 2 m pole. The hook was placed gently around the bird´s neck and used to bring the animal closer to the handler. Once close to the handler, the cormorant´s neck was taken out of the crook by grasping the neck behind the head with one hand and using the second hand to gather the wings up against the body [30]. The camera was then attached to the upper back feathers with Tesa® tape [31] (Fig 1). The cameras were placed facing the head on the dorsal side of the animals (forward-facing). The attachment procedure was completed in less than 5 min, and the birds were released near their nests. Cameras were turned on immediately before deployment and recorded information for a maximum of 80 min (72 ± 17 min) because of limited battery capacity. The cameras were recovered after a single foraging trip. Nests with individuals that were instrumented with a camera were monitored until late in the season and these animals were observed to continue breeding normally after being equipped. Capture, handling and instrumentation procedures were approved by the STyAP and the DFyFS of the Province of Chubut.

### Data collection

Video footage was viewed using Kinovea free video player (http://www.kinovea.org/). Footage included cormorant behavior both at the colony, immediately prior to foraging trips, and during the first hour of foraging. However, for the purposes of this study, we focused only on video segments that were recorded after the animals left the colony to forage.

Behaviors at sea were classified into four categories: flying, washing, floating, and diving. For each flying period, we recorded whether the instrumented animal was alone or accompanied by other seabirds. If the subject was accompanied, the species and number of companions were recorded. Dives were classified into two types: V-shaped and U-shaped. V-shaped dives were characterized by a steep descent angle followed by a steep ascent to the surface and no bottom phase. U-shaped dives had a distinct flat bottom phase preceded by a steep descent phase and followed by an ascent phase. All dive phases were easily distinguished from the video data due to the cormorant´s body position [23].

For each U-shaped dive, we calculated the amount of time the bird spent descending in the water column (define as the interval from the time the camera was being observed to submerge to the time the cormorant was observed to reached the sea floor), the amount of time on the sea floor (the interval from the time the bird reached the sea floor to the time the bird started ascending though the water column), the duration of the ascent phase (the interval from the start of the ascent to the time the camera reached the sea surface), and the post-dive interval (interval at the surface between two consecutive dives). The descent duration was used to calculate the maximum dive depth reached during a particular dive, following the equation: *Maximum depth* = (*Descent duration -*2)/0.7. This equation was constructed with the significant parameters obtained from a mixed effect model that incorporated 992 dives performed by 20 Imperial cormorants instrumented with daily diaries (DD) units [32] with pressure sensors during 2005 and 2006. The accuracy for depth registered by DD units was better than 0.01%. The dives from which the equation was obtained lasted between 2 and 230 s and were performed to depths that ranged between 2 and 55 m [32].

Only dives deeper than 2 m were included in the diving behavior analysis, since shallower dives are known to be associated with activities other than foraging (i.e., washing) [33]. Foraging behavior during the bottom phase of a dive was subdivided into the following activities: (1) capture attempt: a quick turn of the whole body or head and neck or directly when the bird was observed to capture prey, (2) search: the time between two capture attempts, (3) manipulating: the time elapsed between capture and swallowing and (4) swallowing: the time elapsed between prey started and finished being consumed. We also estimated the frequency of capture attempts per bottom time of a dive. The latter was calculated by dividing the number of capture attempts performed during the bottom phase of a particular dive by the total duration of the bottom phase of that dive.

The estimation of cormorant body angle during the bottom phase of dives was determined following the methodology presented by Watanuki et al. [33]. The orientation of the body during the bottom phase was classified as *downward* when the 100% of the image showed the sea floor, *slightly downward* (approximately a 20° angle) when the image showed between 1/4 and 3/8 of water, *horizontal* when the image was 50% water and 50% seabed, and *upward* if only water was visible.

### Microhabitats and prey

Microhabitats associated with each foraging dive were characterized according to sediment type, depth, and organisms present. The sediment type units used were: *predominantly gravel* (the most abundant sediment type around Punta León colony [34]), *rocky*, characterized by the presence of reefs (i.e. isolated small rocky outcrops that extend no longer that a few metres on a flat bottom [35]) and *grasslike* where more than 50% of the seabed was covered by structures such as polychaeta tubes and algae. Captured prey were identified to the lowest possible taxon.

### Luminosity level at the bottom phase

To determine the luminosity level of the microhabitats visited by cormorants, firstly we extracted an image of the bottom phase of each dive. The red, green, and blue (RGB) values for each pixel were obtained using the function readJPEG from the package *jpeg* in R v. 3.0.2 [36]. RGB values were converted to grayscale by using the following function:

Gray=0.299×Red+0.587×Green+0.114×Blue

Once all pixels had been converted to gray scale, we created a luminosity histogram for each image. In this histogram the vertical axis represented the number of pixels (frequency) while the horizontal axis represented the brightness value. Brightness values ranged between 0 (pure black) and 1 (pure white). Following [37], the horizontal axis was divided into 3 zones: 1) a zone with no texture and detail that represented extremely dark or pure white objects in the image (values between 0 and 0.2 and between 0.9 and 1; defined as *Zone 1*), 2) a zone with a limited amount of texture and detail that represented very dark and light objects with some texture (values between 0.2 and 0.3 and between 0.8 and 0.9; defined as *Zone 2*) and 3) a zone with full texture and detail (values between 0.3 and 0.8; defined as *Zone 3*). Each bottom image was classified into one of the previously defined zones by looking at the predominant values of the luminosity histogram. The head of the bird was present in all these images. Special care was taken so that the proportion of pixels occupied by the head was similar between images.

### Data analysis

For each diving parameter the presented grand mean was calculated as the mean of means of each individual. Differences in several diving parameters (i.e. maximum depth, dive, descent, bottom and ascent duration) between cormorants instrumented with cameras and cormorants instrumented with DD during 2005 and 2006 we tested by means of linear mixed effects models (LMM) with Gaussian distribution. These models were run using the *lme* function from the package *lnme* of the open source statistical package R version 3.0.2 [36]. A generalized mixed effect model (GLMM) with Poisson distribution was performed to study the effect of maximum dive depth on the number of capture attempts per dive. The GLMM was run using the *glmer* function from the package *lme4*. In all the mixed effect models bird identity was set as a random factor. Significant effects were detected applying a backward selection procedure and using the *anova* function from the package *stats*.

Statistical analysis were performed with a level of significance of *P*<0.05. Results are shown as the grand mean ± standard error. The median and the range of each calculated variable are also presented.

## Results

Of a total of 33 instrumented cormorants, only 14 left the colony while their cameras were on. Two of these cormorants remained in the coastal area in front of the colony, collecting algae in shallow waters. Consequently, we obtained video footage from 12 foraging animals (Table 1). We recorded a total of 8.1 h of foraging data. Footage at sea lasted between 11.5 and 61.2 min (mean: 40.4 min) (Table 1). Imperial cormorants started their foraging trip in the afternoon between 13:00 and 15:30 h and started diving between 13:20 and 15:36 h (Table 1). The videos showed that cormorants frequently flew with conspecifics during both the outbound flight and the flying periods between dives (Table 1). We obtained video data from 49 foraging dives. Nine of the cormorants (75%) performed exclusively U-shaped dives with a clear bottom phase; only one animal performed exclusively V-shaped pelagic dives. The remaining two animals performed on average 73.3% U-shaped bottom dives and 26.7% V-shaped dives (Table 1).

**Table 1:** General characteristics of the recorded foraging activities performed by 12 male Imperial cormorants (*Phalacrocorax atriceps*) carrying animal-borne video cameras breeding at Punta León, Patagonia, Argentina.

ID	StartTrip	Foraging trip recorded time (min)	Outgoing flight time (min)	Outgoing flight time solitary (min)	Outgoing flight time with con-specifics (min)	Between dives flying time alone (min)	Between dives flying time with con-specifics (min)	First dive	# Dives	# Pelagic V-shaped dives	# Bottom U-shaped dives
1	15:09:14	53.0	7.6	1.3	6.3	7.3	0.0	15:18:09	5	0	5
2	15:28:37	47.7	5.9	0.0	5.9	12.1	0.0	15:35:58	4	0	4
3	14:28:03	11.5	1.6	1.6	0.0	1.0	0.0	14:31:50	4	0	4
4	13:51:00	14.3	3.5	3.5	0.0	0.1	0.0	13:54:37	8	8	0
5	13:43:33	37.0	16.4	9.8	6.6	3.6	0.0	14:04:59	2	0	2
6	13:32:16	33.2	23.6	3.4	20.3	5.1	0.0	13:56:41	1	0	1
7	14:07:02	61.2	21.2	1.2	20.1	11.3	5.0	14:29:07	4	0	4
8	13:28:00	41.5	5.2	1.2	4.0	12.0	12.0	13:34:03	3	0	3
9	12:59:59	42.6	21.1	21.1	0.0	10.5	0.0	13:21:41	2	0	2
10	13:23:12	54.5	10.6	1.8	8.7	7.1	4.4	13:34:54	5	0	5
11	13:43:27	31.6	2.6	2.6	0.0	11.8	0.5	13:46:23	6	2	4
12	13:55:22	57.3	9.4	3.7	5.8	13.2	5.6	14:04:48	5	1	4
Mean ± SE		40.4 ± 4.6	10.7 ± 2.3	4.2 ± 1.7	6.5 ± 2.1	7.9 ± 1.3	2.3 ± 1.1				
Median		42.1	8.5	2.2	5.8	8.9	0.0				
[min—max]		[11.5–61.2]	[1.6–23.6]	[0.0–21.1]	[0.0–20.3]	[0.1–13.2]	[0.0–12.0]				

### Underwater foraging behavior

During the recorded period, the equipped animals performed a total of 38 U-shaped bottom dives (Table 2). The maximum depth reached by Imperial cormorants during these dives was on average 44.3 ± 3.8 m (Table 2). Birds spent on average 172.1 ± 13.1 s underwater (Table 2). When splitting the time underwater into descent and ascent phases, we found that the descent phase had a mean duration of 33.0 ± 2.7 s, while the ascent phase had an average duration of 31.7 ± 2.5 s (Table 2). No difference was found in the duration of these diving phases between birds equipped with cameras (present study) and birds equipped with DD during previous seasons (LMMs; L ratio = 0.4 and P = 0.5 for the maximum depth, L ratio = 0.4 and P = 0.6 for the dive duration, L ratio < 0.01 and P = 0.99 for the descent phase, L ratio = 0.08 and P = 0.8 for the ascent phase).

**Table 2:** Characteristics of the dives and capture events of 12 male Imperial cormorants (*Phalacrocorax atriceps*) carrying animal-borne video cameras breeding at Punta León, Patagonia, Argentina.

ID	# Bottom U-shaped dives	Meandive duration (s)	Mean descent duration (s)	Mean bottom duration (s)	Mean bottom search time (s) (%)	Mean bottom capture time (s) (%)	Mean ascent duration (s)	Mean maximum depth (m)	Total number of capture attempts	Mean capture attempts per dive	Mean capture attempts duration (s)	Mean number of capture events per bottom time (s^-1^)
1	5	197.6	29.6	140	116.6 (83)	22.6 (16.4)	28	39.4	36	7.2	3.1	0.05
2	4	169	34.8	135.5	128.8 (95)	4 (3)	31.5	46.8	13	3.3	1.2	0.02
3	4	64.3	10	44.3	49 (92)	1 (3)	10	11.4	2	1	1	0.01
4	0	-	-	-	-	-	-	-	-	-	-	-
5	2	220.5	38	144	127 (94.8)	9 (7)	38.5	51.4	4	4	2.3	0.01
6	1	148	29	91	80 (87.9)	9 (10)	28	38.6	3	3	3	0.03
7	4	176	42	100.8	91.8 (91.1)	9 (7)	40.7	57.1	7	3.5	3	0.01
8	3	175.7	33.3	109.7	100.3 (91.0)	9 (9)	32.7	44.8	6	2	4.7	0.02
9	2	221	37.5	147	136.5 (92.7)	5.5 (4)	36.5	50.7	4	2	2.8	0.01
10	5	201.4	40	124.6	121.4 (97.6)	4 (3)	36.8	54.3	8	2	2	0.01
11	4	149.8	30.3	88.3	83.8 (95.9)	5 (4)	31.3	40.4	7	2.3	2.1	0.02
12	4	169.8	39	96.5	85.8 (88.2)	9.5 11)	34.3	52.9	10	2.5	1.4	0.03
Mean ± SE		172.1 ± 13.1	33.0 ± 2.7	111.1 ± 9.4	101.9 ± 8.0	8.0 ± 1.7	31.7 ± 2.5	44.3 ± 3.8	9.1 ± 2.9	3.0 ± 0.5	2.4 ± 0.3	0.02 ± 0.004
Median		175.7	34.8	109.7	100.3 (92.6%)	9.0 (6.7%)	32.7	46.8	7.0	2.5	2.3	0.02
[min—max]		[64.3–221.0]	[10.0–42.0]	[44.3–147.0]	[49.0–136.5]	[1.0–22.6]	[10.0–40.7]	[11.4–57.1]	[2.0–36.0]	[1.0–7.2]	[1.0–4.7]	[0.01–0.05]

**Fig 2:**
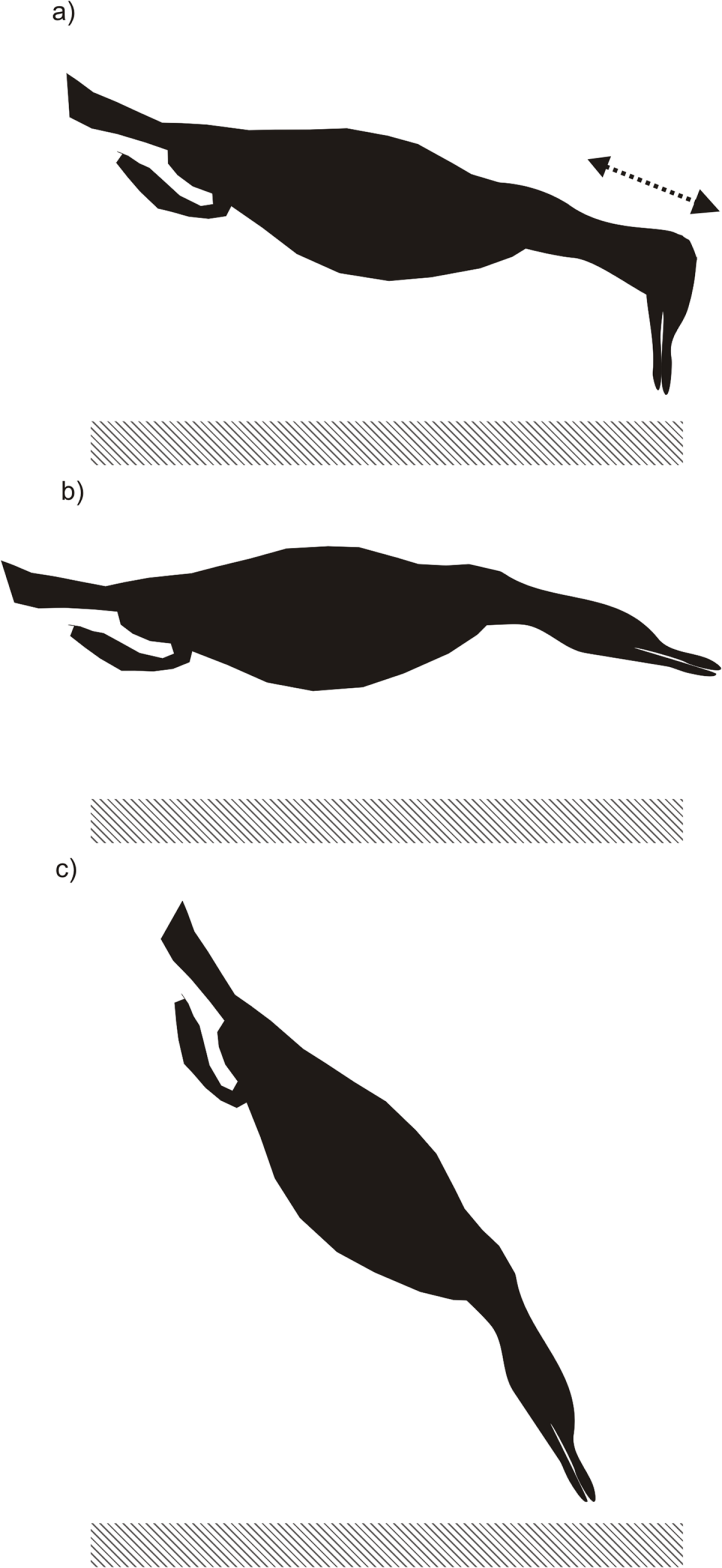
Body position of Imperial cormorants while foraging along the seabed. Diagram showing the body position a) while searching for prey along the sea floor in the *predominantly gravel* microhabitat (the black arrow represents the extension and contraction of the neck as the birds forages) b) while searching along the seafloor in the *rocky* microhabitat and c) while capturing and consuming prey during the bottom phase of dives. Striped rectangles indicate the sea floor.

During the U-shaped benthic dives, the average amount of time spent foraging along the sea floor was 111.1 ± 9.4 s (Table 2). No difference was found in the amount of time spent foraging along the sea floor between cormorants equipped with cameras (present study) and cormorants equipped with DD during previous seasons (LMM, L ratio = 1.2 and P = 0.3). Video images showed that birds did not necessarily keep their body horizontal while they were at the bottom, but inclined it slightly downward (Fig 2A). The heads of the animals were always visible in the videos. Thus, it was clear that in a majority of the dives (76.3%), foraging birds moved their heads constantly forward and backward by extending and contracting their necks while traveling on the seafloor (Fig 2A). During the bottom phase of most dives, birds kept their necks at an angle of approximately 90° to the seafloor, with their heads facing downwards (Fig 2A). In some dives (7.3%), birds swam along the seafloor almost in a horizontal position, keeping their heads and necks extended for most of the time (Fig 2B).

**Fig 3:**
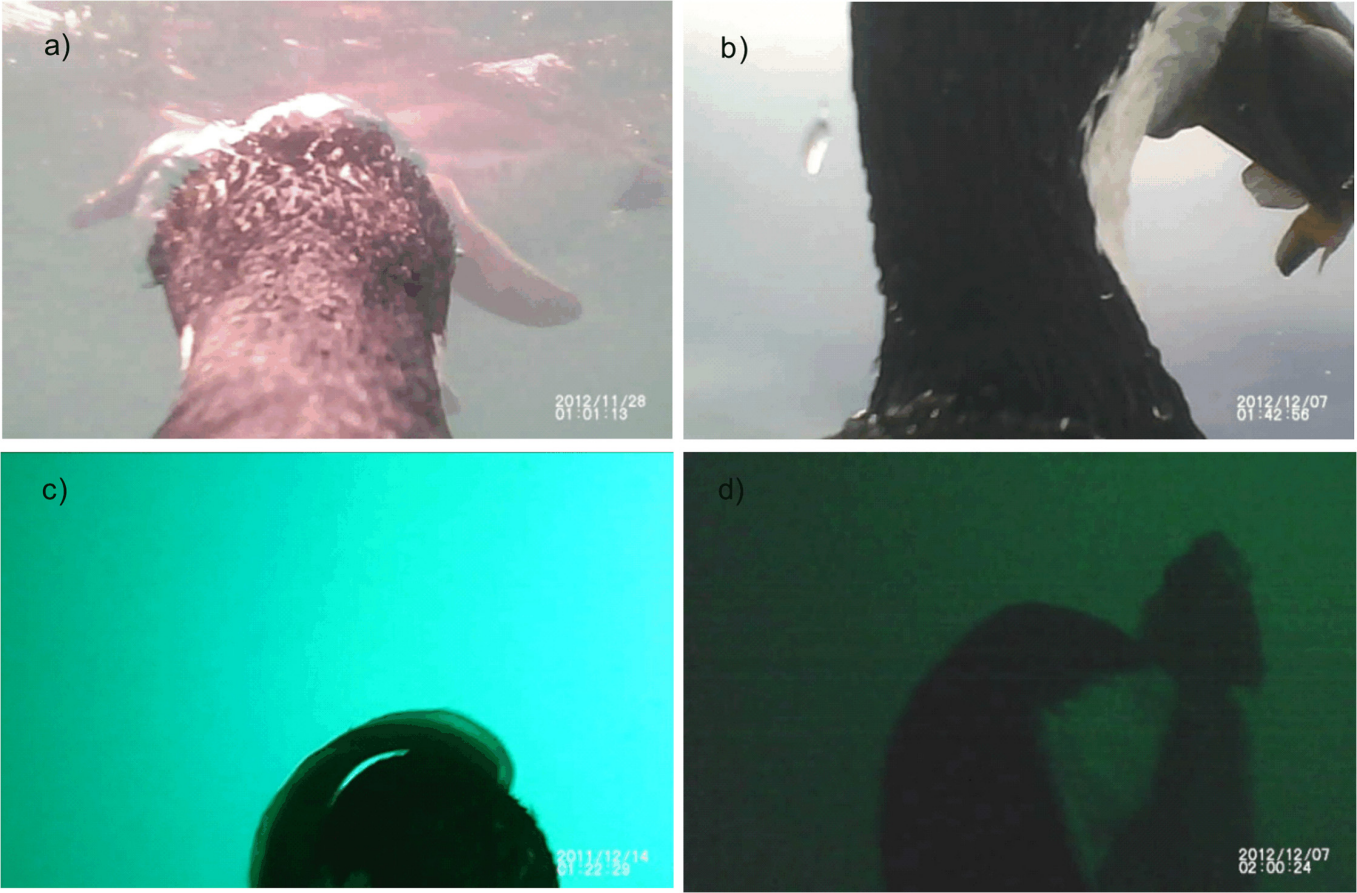
Still frames taken from Imperial cormorants (*Phalacrocorax atriceps*) videos. Representative examples showing a) the presence of a penguin in the ascent phase of a dive, b) a bird at the sea surface swallowing an Argentine seabass, c) a bird ascending through the water column with a curk-eel, and d) a bird carrying a Channel bull blenny. Each picture date and time is not the real one but the one provided by the camera.

In only one dive (2.9%), another cormorant was registered in the field of view of the camera, while in two occasions (4.1%) a Magellanic penguin (*Spheniscus magellanicus*) was recorded in the ascent phase of a dive (Fig 3A).

#### Capture methods and strategies

We calculated the percentage of time searching and attempting to capture prey for 36 out of 38 U-dives (in the remaining two dives video images were too dark to recognize behaviours). Male Imperial cormorants spent on average 92.6% of their time at the bottom searching for food and 6.7% capturing prey. The rest of the bottom time (less than 1%) involved manipulating and swallowing prey.

We did not see any evidence that cormorants probed into the substrate with their bills. Rather birds seemed to detect prey at short distances and employ a prey-flushing strategy. In other words, cormorants exploited the escape response that they triggered on prey.

During capture attempts, cormorants made quick turns and maintained their body in a vertical position facing downwards (Fig 2C). Capture attempts had a mean duration of 2.4 ± 0.3 s (Table 2). The average number of capture attempts per dive was 3.0 ± 0.5 while the average number of capture attempts per bottom time was 0.02 ± 0.004 s ^-1^ (Table 2). Maximum dive depth was not observed to have an important effect on the number of capture attempts per dive (GLMM, X^2^ = 0.02, P = 0.9).

In those capture attempts where the capture, manipulation, and swallow events took place underwater and could be clearly distinguished from one another (n = 6), the manipulation time ranged between 1 and 17 s, while the amount of time spent swallowing prey ranged between 1 and 2 s. In three other occasions, cormorants were observed to capture prey in the last few seconds of the bottom phase, subsequently carrying it in the bill to the surface. Once on the surface, the amount of time animals spent manipulating prey ranged between 3 and 22 s. We should note here that because Imperial cormorants mostly consume small and cryptic prey (see next section), manipulation and swallowing times could only be determined when large, visible prey were captured. The success rate where prey were observed being capture was of 100%.

#### Prey type

Video images revealed that cormorants are almost exclusively benthic feeders; we only observed one occasion in which an animal captured and swallowed a Patagonian red shrimp (*Pleoticus muelleri*) during the ascent phase. Although we were able to clearly observe the cormorants feeding, it was difficult to detect and identify specific prey items by using the video cameras. We were able to identify prey species on only four occasions. Surprisingly, benthic fish (*Riberoclinus eigenmanni*) and toadfish (*Thiathalassothia argentina*) were not found in any of the videos despite their prevalence in the diet of Imperial cormorants at Punta León [22]. We attribute this finding to three factors: (1) the small size of these two prey species, (2) their camouflage, and (3) some of the recorded dives were performed under very low luminosity conditions.

The four events in which prey were recognized corresponded to birds returning to the surface with a prey item in its bill. Cormorants were observed to have captured: Argentine seabass (*Acanthistius brasilianus*) in two cases (Fig 3B), a curk-eel (*Raneya brasiliensis*) in one case (Fig 3C), and what was likely a channel bull blenny (*Cottoperca gobio*) in one case (Fig 3D). Prey items captured and consumed at the bottom could not be identified.

#### Microhabitat use and luminocity level at the bottom phase

**Fig 4:**
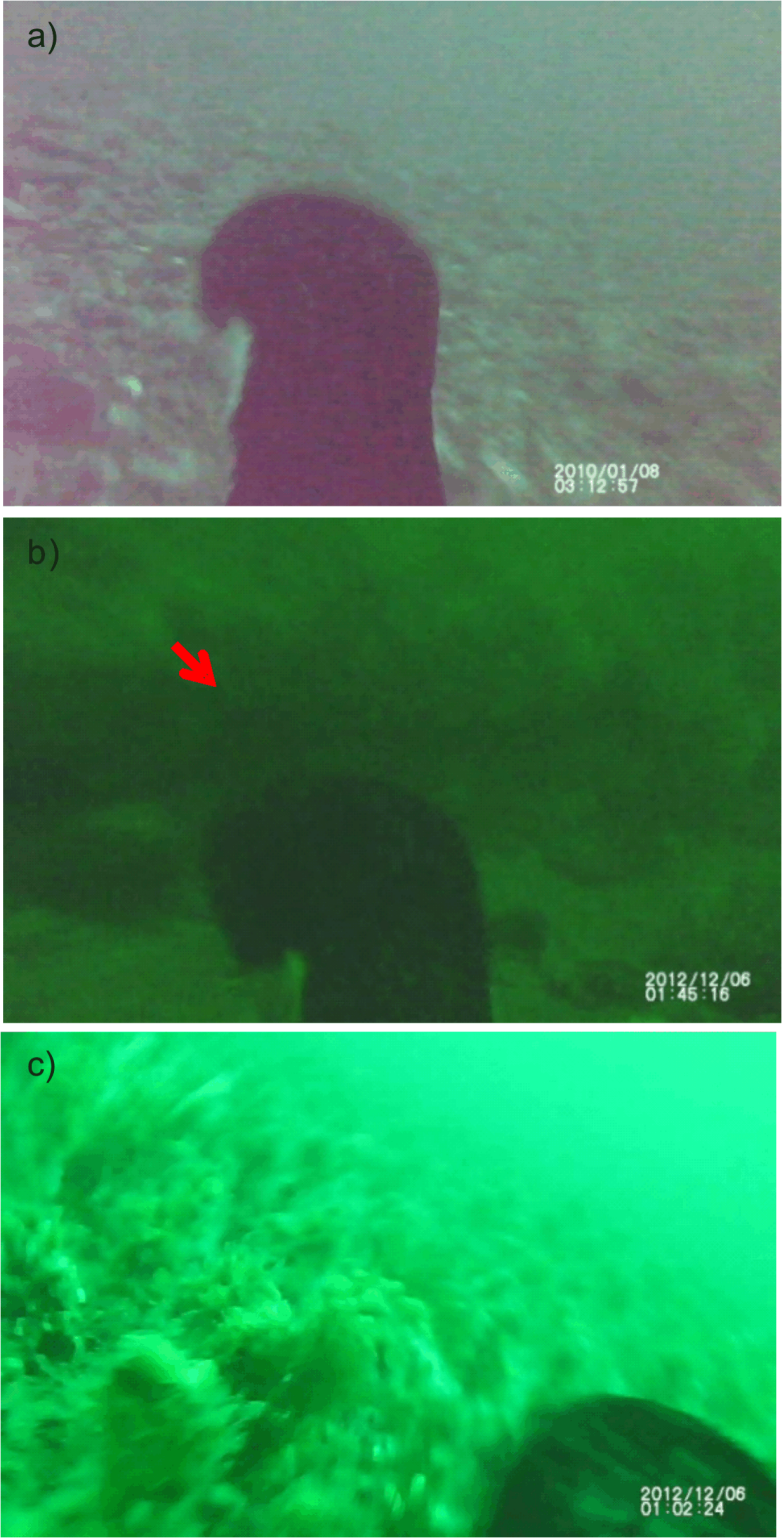
Still frames taken from Imperial cormorants (*Phalacrocorax atriceps*) videos. Representative examples showing a) a bird in searching for prey in the type 1 habitat, b) a bird foraging in a rocky reef (indicated by a red arrow), and c) a bird looking for food in the type 3 habitat. Each picture date and time is not the real one but the one provided by the camera.

Instrumented cormorants foraged at three different sea bottom habitats. Most of the dives (77.8%) reached depths between 20 and 60 m on a seafloor composed of a matrix of predominantly gravel that also contained broken mollusk shells, and pebbles (hereafter *gravel*). This habitat supported patches of anemones, gorgonians, snails, and algae (Fig 4A). In 19.4% of the dives, animals visited *rocky* habitats (Fig 4B). Dives over this substrate were between 10 and 55 m in depth. Finally, in one dive (2.8%) performed at a depth of 35 m, a cormorant was observed to explore a *grasslike* habitat (Fig 4C).

The way that cormorants searched for food differed between habitats. During the bottom phase of dives to *gravel* and *grasslike* substrates, birds maintained their body inclined slightly downward and moved their heads constantly forward and backward (Fig 2A & 2B). On dives to *rocky* bottoms, birds clearly swam in a horizontal position (see above section on underwater behavior), navigating between rocks and foraging by inserting the neck and head between reef ledges (Fig 2C).

**Fig 5:**
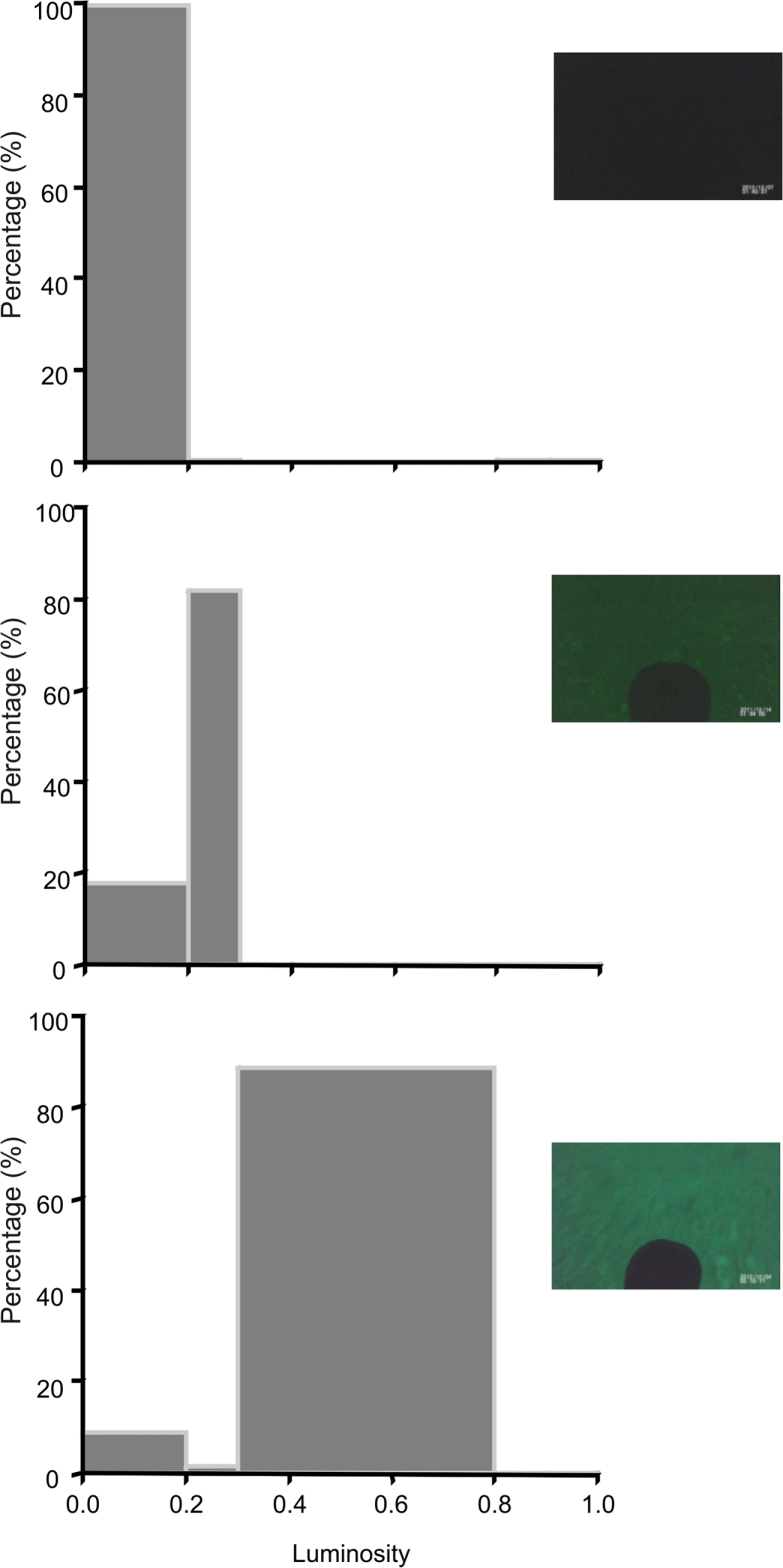
Frequency distribution (%) of pixel brightness values. Representative examples of a) a picture classified as belonging to *Zone 1*, b) a picture classified as belonging to *Zone 2* and c) a picture from *Zone 3*.

Of 38 images, one for the bottom phase of each U-shaped dive, 37% was classified as belonging to *Zone 1* (Fig 5A), 45% to *Zone 2* (Fig 5B) and 18% to *Zone 3* (Fig 5C).

## Discussion

### Device effects

It is generally accepted that in order to avoid disturbances caused by the attachment of external devices, the weight of the attached instrument should not exceed 3% of the birds’ body mass [38, 39] and should be placed in the lower back of the body in order to reduce drag as much as possible [40]. Even though the video cameras employed in the present study did not exceed the 3% threshold they were placed in the upper back of the animal so we cannot rule out the possibility that they might have had an impact on cormorant foraging behavior. However, we point out that the diving parameters presented in this study did not differ from previous data obtained by means of DD units [32]. These units were lighter than cameras and placed on the lower back of the body.

### Underwater foraging behavior

Even though Imperial cormorants from Punta León frequently flew with conspecifics to their foraging grounds, we did not detect any evidence that they fed in group. Solitary feeding might be favored by birds feeding on sparse and/or cryptic benthic prey in rocky and gravel habitats, where the chances of encountering and capturing other nearby prey items are small. In such cases, Imperial cormorants might rely on underwater topographic cues to find prey, similar to other species in this genus (e.g., *P*. *melanogenis*) [41]. These types of cues allow animals to memorize the location of rich foraging grounds and return to those areas on successive trips [18, 42].

Our video data on cormorant underwater body orientation clearly support previous findings obtained by the use of accelerometers [23, 43]. To summarize, cormorants descended along the water column with their bodies pointing fully downward, moved along the sea floor with their bodies pointing slightly downward, and then ascended to the surface with their bodies pointed upward. However, contrary to data obtained with accelerometers [43], we did not detect any evidence of body rolling during the bottom phase of dives. This discrepancy between video and accelerometer data could be attributed to the high sensitivity of the accelerometers. Future studies using both types of devices simultaneously will help to elucidate this particular issue.

The frequency of capture attempts and the speed reached on pursuits is critical for air breathing predators [44, 45]. Swimming power requirements increased as a cubed function of the speed [44] and this translates in more oxygen consumption and a reduction in the time cormorants may spend on the bottom searching for prey. Our results showed that once on the seabed, animals spent most of their time searching for prey and made quick and short capture attempts. There was no evidence of birds engaging in fast and long pursuits. This information agrees with previous results obtained by the instrumentation of animals with inter-mandibular angle sensors [20]. Moreover it supports the fact that cormorants employ a brief short-distance pursuit technique [46]. Short pursuits could be a way of reducing the amount of energy expended in capturing prey; however the speed of capture attempts should be measured in order to confirm this. Due to the important intersexual differences in the foraging behavior of Imperial cormorants [32, 47, 48] it would also be interesting to compare both sexes capture rates and techniques. Males and females exploit different depths [32, 47] and prey [47] thus they represent a good model to test the effect of buoyancy and prey type on the fine scale foraging behavior of a seabird diving species. The fact that females depart very early in the morning [28] and perform their first dives under low luminosity levels precluded us from establishing intersexual comparisons.

### Prey type and microhabitat use

Even though the videos only allow us to detect and identify large prey species (consequently restricting our calculations on prey capture and handling time), they undoubtedly represent the most comprehensive data so far collected on microhabitat use by Imperial cormorants while foraging. Our study shows that cormorants use at least three different microhabitats of varying biodiversity and physical features. The most commonly used microhabitat was a gravel bottom characterized by low levels of marine life, followed by an area with numerous reefs and rocky outcrops. In one instance, we observed cormorants diving to a bottom covered by polychaete tubes and algae. Because the cormorants overwhelmingly used one microhabitat over the other two, we were unable to make statistical comparisons of their diving behavior (e.g., diving depth, bottom duration, capture attempts) across microhabitats. However, in a general sense, cormorant body inclination and foraging technique were quite different between the two most frequently visited habitats suggesting different foraging strategies among habitats.

### Visual ecology

The amphibious lifestyle of cormorants imposes important optical challenges because aquatic optical requirements differ from those in air [49]. Corneal refractive power is lost underwater and this creates a reduction in the size of visual fields [50, 51]. Thus, predators hunting underwater should have visual adaptations if they rely on sight to capture prey. However, although cormorants have been generally regarded as visually-guided pursuit-dive predators, a recent study has demonstrated that they have poor ability to resolve visual detail underwater and are only capable of detecting prey visually at short distances (less than 1 m) [46]. Our video data strongly support the idea that their efficient hunting involves the use of specialized foraging techniques to compensate for their poor underwater vision. This involves a prey-flushing strategy that triggers an escape response from prey, making them more visually conspicuous. Cormorants then employ brief short-distance pursuit and/or rapid neck extension to capture flushed prey at short range [46]. The latter was supported by the observation of quick and short capture attempts and the absence of long and fast pursuits.

While foraging along the seafloor, cormorants were frequently observed to move their head constantly forward and backward. This rhythmic movement of the head called “head-bobbing” is observed in many avian species [52, 53] and has functional implications for vision (e.g. [54]). The hold phase of head-bobbing allows birds to stabilize retinal images while the thrust phase permits to obtain depth information (e.g. [54, 55, 56]). Although most of the studies about head-bobbing have been performed on walking species, a recent work suggests it would also play an important role in stabilizing underwater vision [57]. Thus, the head bobbing movement in combination with other characteristics such as a long and narrow binocular field [24] supports the fact that even though these animals are not pursuit foragers, they are visually guided predators.

Cormorants´poor visual acuity is further reduced under low light conditions [46]. Several authors have proposed that in turbid and dark water environments, cormorants must employ tactile and/or acoustic foraging strategies [26, 58, 59]. Although we did not measure light, our data revealed that while foraging along the seafloor cormorants frequently visit low luminosity environments. Luminosity levels would be even lower than the ones reported here from a 57 m depth dive if we consider that male Imperial cormorants dive up to 100 m depth [60]. Thus, even though we found no evidence of Imperial cormorants locating prey by touch, in agreement with previous studies [25, 58, 59] we believe these animals would locate prey with other sensory systems in addition to sight, especially on their deepest dives.

## Conclusions

Use of animal-borne video cameras corroborated several aspects of Imperial cormorants’ foraging behavior previously determined by other electronic devices. At the same time, the use of cameras offered new insights into Imperial cormorants foraging behavior and seems to be a powerful tool for investigating how these predators capture prey. In addition, cameras provide information about the biological and physical characteristics of the underwater habitats where cormorants foraged. In agreement with Martin et al. [24] and White et al. [27], the present study also supports the hypothesis that cormorants use a prey-flushing instead of a prey-pursuit strategy. As recently observed in greebes [57], the head-bobbing movements observed while cormorants swam along the seafloor would allow them to stabilize underwater vision, however how this animals find prey under low luminosity conditions remains still a question. Future research approaches should consider the simultaneous use of cameras, GPS loggers, depth recorders, and accelerometers for a better comprehension of this predator’s underwater visual and hunting ecology.
